# Comparative Analysis of Plastid Genomes in the Non-photosynthetic Genus *Thismia* Reveals Ongoing Gene Set Reduction

**DOI:** 10.3389/fpls.2021.602598

**Published:** 2021-03-16

**Authors:** Sophia V. Yudina, Mikhail I. Schelkunov, Lars Nauheimer, Darren Crayn, Sahut Chantanaorrapint, Michal Hroneš, Michal Sochor, Martin Dančák, Shek-Shing Mar, Hong Truong Luu, Maxim S. Nuraliev, Maria D. Logacheva

**Affiliations:** ^1^Faculty of Biology, Lomonosov Moscow State University, Moscow, Russia; ^2^Institute for Information Transmission Problems, Moscow, Russia; ^3^Joint Russian-Vietnamese Tropical Scientific and Technological Center, Hanoi, Vietnam; ^4^Skolkovo Institute of Science and Technology, Moscow, Russia; ^5^Australian Tropical Herbarium, James Cook University, Cairns, QLD, Australia; ^6^Division of Biological Science, Faculty of Science, Prince of Songkla University, Hat Yai, Thailand; ^7^Faculty of Science, Palacký University Olomouc, Olomouc, Czechia; ^8^Centre of the Region Haná for Biotechnological and Agricultural Research, Crop Research Institute, Olomouc, Czechia; ^9^Ying Wa College, Hong Kong, China; ^10^Southern Institute of Ecology, Graduate University of Science and Technology, Vietnam Academy of Science and Technology, Ho Chi Minh City, Vietnam

**Keywords:** *Thismia*, plastid genome, non-photosynthetic plants, genome reductive evolution, mycoheterotrophy

## Abstract

Heterotrophic plants provide intriguing examples of reductive evolution. This is especially evident in the reduction of their plastid genomes, which can potentially proceed toward complete genome loss. Several milestones at the beginning of this path of degradation have been described; however, little is known about the latest stages of plastome reduction. Here we analyze a diversity of plastid genomes in a set of closely related non-photosynthetic plants. We demonstrate how a gradual loss of genes shapes the miniaturized plastomes of these plants. The subject of our study, the genus *Thismia*, represents the mycoheterotrophic monocot family Thismiaceae, a group that may have experienced a very ancient (60–80 mya) transition to heterotrophy. In all 18 species examined, the plastome is reduced to 14–18 kb and is highly AT-biased. The most complete observed gene set includes *accD*, seven ribosomal protein genes, three rRNA, and two tRNA genes. Different clades of *Thismia* have undergone further gene loss (complete absence or pseudogenization) compared to this set: in particular, we report two independent losses of *rps2* and *rps18*.

## Introduction

Heterotrophic plants are an intriguing ecological group of organisms. Most plants, which obtain carbon compounds through fixation of atmospheric carbon dioxide, use the energy of light (photosynthesis), but heterotrophs obtain fixed carbon from other plants or fungi (Leake, 1994; Těšitel, 2016). The heterotrophic way of life is associated with highly modified morphology, physiology, and genome organization (Merckx, 2013; Yuan et al., 2018). Plastomes of heterotrophic plants show considerable gene loss, size reduction (the plastome size decreases up to 11 vs. 110–200 kb in autotrophic plants) and usually elevated substitution rates [reviewed in Graham et al. (2017)]. However, in most non-photosynthetic plants, plastomes are retained because they perform vital functions unrelated to photosynthesis (Barbrook et al., 2006). The initial stages of modification of the plastid genome in heterotrophic plants have been broadly studied in different lineages (Barrett et al., 2014, 2018, 2019; Samigullin et al., 2016); they consist of rapid and coordinated loss or pseudogenization of photosynthesis-related genes. The variation of the overall plastome structure in heterotrophs of recent origin is typically minor. The middle stages of plastome reduction have been also extensively studied in both parasitic and mycoheterotrophic plants (Wicke et al., 2016; Lallemand et al., 2019; Chen et al., 2020). These studies have shown that the patterns of gene loss in general follow the model of Barrett and Davis (Barrett and Davis, 2012). This model postulates that the first to be lost are the genes related to photosynthesis and the last – genes related to the translation (ribosomal and transfer RNAs, ribosomal proteins) and other “housekeeping” functions. In contrast to initial and middle stages of plastome reduction, species with a high level of reduction have never been characterized in a phylogenetic context with a dense taxonomic sampling. Although there are many reports of single or several highly reduced plastomes (e.g., Schelkunov et al., 2015; Bellot and Renner, 2016), these studies have been focused mostly on the reduction itself, and on the similarity (or difference) of reduction patterns in unrelated groups. The model thus lacks resolution for the group of “the last ones to be lost” genes – it is clear that they can be also lost but the tendencies of the loss are not completely clear. To better understand the steps that led to the highly reduced plastome structure, we focused on patterns of variation of the genomes and gene sets in a group of closely related species, in the genus *Thismia* (Thismiaceae, Dioscoreales). This is an exclusively heterotrophic genus (the family is also fully heterotrophic) comprising about 90 species that are widely distributed in tropical and subtropical regions of Asia, Australasia, and America, but extending to some temperate areas. Representatives of *Thismia* are tiny herbs, remarkable for their unusual appearance, curious flower morphology and highly specialized pollination biology (Merckx, 2013; Guo et al., 2019; Dančák et al., 2020a; Shepeleva et al., 2020; Nuraliev et al., 2021). Thismiaceae may represent one of the most ancient mycoheterotrophic groups of plants: according to Merckx et al. (2010), they appear to have originated in the late Cretaceous, although Givnish et al. (2018) infer a much more recent origin. We assembled complete and nearly complete plastomes for 17 species of *Thismia* and compared them with the previously characterized plastome of *Thismia tentaculata* K.Larsen & Aver (Lim et al., 2016). Using this expanded dataset of *Thismia* plastomes we identified changes in structure and gene content of the plastid genome, and performed phylogenetic reconstruction based on the plastid gene sets.

## Materials and Methods

### Sequencing and Assembly

We included data for 18 species of *Thismia*, 17 of which were generated *de novo* here, with each species represented by a single specimen (see Supplementary Table 1 for material information). Sequences for *Thismia tentaculata* (KX171421), *Dioscorea elephantipes* Engl. & Drude (NC_009601), *Burmannia disticha* L. (NC_036661), *Burmannia coelestis* D. Don (NC_036660), *Tacca chantrieri* André (KX171420), and *Tacca leontopetaloides* (L.) Kuntze (NC_036658), used in the phylogenetic and comparative analyses, were obtained from GenBank (accession numbers in parentheses). Total genomic DNAs were extracted from fresh, herbarium, and silica gel-dried material using the protocol of Doyle and Doyle (1987). DNAs were fragmented using a Covaris S220 sonicator (Covaris, United States) with the following settings: time 40 s, peak power 175 W, duty cycle 10%. Genome libraries were prepared using a method based on ligation of partially double-stranded adapters to adenylated DNA (Bentley et al., 2008) with a NEBNext Ultra II DNA kit (New England Biolabs, United States). Libraries were sequenced using several Illumina platforms: HiSeq, MiSeq, and NextSeq (Illumina, United States). For the data generated in this study (except for *Thismia mucronata* Nuraliev, *Thismia hawkesii* W.E.Cooper, and *Thismia lanternata* W.E.Cooper, see below), reads were trimmed and assembled using CLC Genomics Workbench v. 7.0.3 with the following parameters: word size and bubble size = default, minimal contig length = 1000, mismatch cost = 2, insertion cost = 3, deletion cost = 3, length fraction = 0.98, and similarity fraction = 0.99. Plastid contigs were selected from the total set of contigs based on their coverage and similarity to the *T. tentaculata* plastome. All candidate contigs were checked using nucleotide-based BLAST searches (Camacho et al., 2009) to exclude mitochondrial and high-copy nuclear genome fragments. The resulting contigs for each species were searched for overlaps between ends and then joined. The borders of the inverted repeat (IR) and single copy regions were identified by manual searching and alignment of reads spanning possible junction points.

The plastomes of *T. hawkesii* and *T. lanternata* were assembled using Geneious Prime^1^, then reads were mapped onto the plastome of *T. tentaculata* as reference and the retrieved contig sequences were extended by remapping the reads onto them until only three contigs remained (presumed to correspond to the single copy and inverted repeat regions of the plastome). These contigs were concatenated to obtain the full plastome sequence. A final remapping of reads was performed to ensure the plastome sequences were recovered fully and without overall assembly errors.

For the plastome of *T. mucronata*, which was the first plastome to be assembled and the only one sequenced using longer reads (255+255) on MiSeq, we used an alternative approach. Its reads were trimmed using Trimmomatic 0.32 (Bolger et al., 2014), removing both adapters and read regions with low quality. We then joined overlapping reads using fastq-join from the ea-utils 1.1.2 set of tools (Aronesty, 2013), requiring reads to merge if they overlapped by at least 30 bp with at least 80% sequence similarity. To filter for nuclear sequences, we used Kmernator 1.2.0^2^ to remove all 31 bp k-mers that had sequencing coverage less than 30X. We assembled resulting single reads using Newbler 2.6, an assembler initially developed for 454 (Roche, Switzerland) data but which we found to be suitable for assembling relatively long Illumina reads. Contigs of plastid origin were then selected using BLAST and extended using Mapsembler 1.3.21 (Peterlongo and Chikhi, 2012) using a k-mer size of 75 bp. For *T. mucronata*, we checked IR borders using polymerase chain reaction and further visualization of PCR products on the agarose gel for length estimation (primer pairs Thism-C2-F/Thism-C1-R for LSC/IRa, Thism-C1-F/Thism-C7+C6-R for IRa/SSC, Thism-C7+C6-F/Thism-C1-F for SSC/IRb, and Thism-C1-R/Thism-C2-R for IRb/LSC, for primer sequences and locations see Supplementary Table 2).

For all species partial and complete plastid genome sequences were annotated using DOGMA (Wyman et al., 2004) and Chlorobox (Tillich et al., 2017) using relaxed parameters for protein and RNA identity. Identification and annotation of tRNA and rRNA genes was also performed using online tRNAscan-SE (Chan and Lowe, 2019) and RNAmmer 1.2 (Lagesen et al., 2007). The annotation was then checked and corrected manually based on the alignment with orthologous genes from *T. tentaculata* and other relative species. For the visualization of plastid genome maps we used OGDraw (Lohse et al., 2007).

For transcriptome sequencing, we extracted RNA from above-ground parts of the living plant of *Thismia puberula* Nuraliev using RNeasy Mini kit (Qiagen, Netherlands). RNAs from different above-ground parts of the plant were pooled in equal amounts and used for further sample preparation. For the removal of ribosomal RNA we used Ribo-Zero Plant kit (Illumina, United States). The library was prepared using NEBNext Ultra II RNA sample preparation kit (New England Biolabs, United States) according to the manufacturer’s instructions, except for the duration of fragmentation time (1 min). For sequencing, we used NextSeq instrument (Illumina, United States) in 150 bp paired-end mode. Assembly was performed using CLC Genomics Workbench with the settings described above for plastid genomes. In order to estimate expression levels and to detect RNA editing we mapped the reads against the plastome of *T. puberula*. The parameters for mapping were as follows: mismatch cost 2, insertion cost 3, deletion cost 3, length fraction 1.0, similarity fraction 0.99.

### Phylogenetic Inference

We aligned genes from all *Thismia* plastid genomes and an outgroup in the same order (*D. elephantipes*, Dioscoreaceae), for protein-coding genes using TranslatorX 1.1 with MAFFT 7.402 (Abascal et al., 2010; Katoh and Standley, 2013), and for rRNA- and tRNA-coding genes using MAFFT 7.402. We pruned alignments using Gblocks 0.91b (Castresana, 2000), with default parameters except that columns were removed based on the proportion of sequences with gaps only if there were gaps in at least 50% of sequences. By default, a gap in a single sequence is enough for Gblocks to remove a column. After pruning, separate alignments for each gene were concatenated together. We inferred phylogenetic trees using three methods (1, 2 are maximum likelihood methods, 3 is a Bayesian inference method):

To infer the alignment partitioning and the nucleotide substitution models that are optimal for phylogenetic tree building, we used IQ-TREE 2.0.6 (Minh et al., 2020) with the extended model selection option (“-m MFP”). Based on the Bayesian information criterion, IQ-TREE suggested to split the alignment into the following three partitions:

1. The genes *rrn16* and *rrn23* with the substitution model GTR+F+R3. “GTR” is the general time reversible model. “F” means base frequencies were calculated directly from the multiple alignment. “R3” means the FreeRate model of substitution rates with 3 site categories.
2. The genes *accD*, *rpl2*, *rps12*, *rps18*, *rps2*, *trnE-UUC*, *trnfM-CAU*, and *rrn5* with the substitution model TVM+F+G4. “TVM” is a model similar to GTR but with the A→G substitution rate equal to the C→T substitution rate. “G4” is the Gamma model of substitution rates with 4 site categories.
3. The genes *rps3*, *rps4*, and *rps8* with the substitution model TPM2+F+G4. TPM2 is a model with the A→C substitution rate equal to the A→T substitution rate, the A→G substitution rate equal to the C→T substitution rate, and the C→G substitution rate equal to the G→T substitution rate.

We inferred phylogenetic trees using three methods (1, 2 are maximum likelihood methods, 3 is a Bayesian inference method):

1. IQ-TREE 2.0.6. Bootstrap support values for the maximum likelihood tree were calculated using 1000 iterations of the UFBoot algorithm (Hoang et al., 2018).
2. RAxML 8.2.12 (Stamatakis, 2014) with 20 starting maximum parsimony trees. Bootstrap support values for the maximum likelihood tree were calculated using the Rapid Bootstrap algorithm with the number of bootstrap pseudoreplicates determined by RAxML automatically (the “autoMRE” option). RAxML has a scarcer choice of nucleotide substitution models than IQ-TREE, particularly it has no analogs of IQ-TREE’s TVM, TPM2, and R3. For this reason, we used GTR+F+G4 models, which are similar to the models advised by IQ-TREE, for each of the three partitions.
3. MrBayes 3.2.7 (Ronquist et al., 2012) with four Markov chains, each of 2,500,000 generations, and sampling frequency of 500 generations. Majority-rule consensus trees were calculated after excluding the first 25% of samples. The model choice in MrBayes is scarcer than in IQ-TREE. Particularly, similar to RAxML, it isn’t capable of using TVM, TPM2, and R3. For this reason, we used GTR+F+G4 models for each of the three partitions.

We reconstructed the history of gene losses by Mesquite 3.51 (Maddison and Maddison, 2018) using the maximum parsimony method.

### Selection Analysis

To perform selection analyses that consider the ratio of non-synonymous to synonymous substitutions (dN/dS), we aligned protein-coding genes as described in the “Phylogenetic Inference” section, but instead of using *D. elephantipes* as the outgroup, we used *B. disticha*, *B. coelestis*, *T. chantrieri*, and *T. leontopetaloides*. All three methods of tree construction (see above) resulted in trees with the same topology, which was assumed for the selection analysis. The dN/dS analysis was performed using PAML 4.9 (Yang, 2007). An unrooted topology provided to PAML, thus the placement of *Thismia*, *Burmannia*, and *Tacca* clades with respect to each other is not relevant. For each gene, we ran PAML two times:

1. In the first calculation, one dN/dS value was allowed for *Thismia* branches, a second dN/dS value for branches of photosynthetic species, and a third for the branch where photosynthesis is assumed to have been lost (i.e., the stem lineage of *Thismia*; in unrooted terms, the branch connecting the clade of *Thismia* to the *Burmannia* and *Tacca* clades). The third dN/dS value was on the branches of *Burmannia* and *Tacca*.
2. In the second calculation, one dN/dS value was allowed for the branch where photosynthesis was lost and the other dN/dS value for all other branches.

A separate value for the branch where photosynthesis was lost is desirable because that branch can be attributed neither to non-photosynthetic nor to photosynthetic plants.

*P*-values for the hypothesis of dN/dS difference between non-photosynthetic and photosynthetic species were calculated for each gene separately using the likelihood ratio test. We performed the multiple hypothesis testing correction using the method of Benjamini and Hochberg (1995) to account for the fact that each gene required a separate test.

We used PAML with the following parameters: runmode = 0, seqtype = 1, CodonFreq = 2, icode = 0, model = 2, NSsites = 0, fix_kappa = 0, kappa = 2.0, fix_omega = 0, omega = 0.5, fix_blength = 0, cleandata = 0.

The removal of alignment columns with gaps may influence the dN/dS calculations, because indels likely occur in regions with neutral or positive selection and less likely to occur in regions with negative selection. To evaluate the influence of gap removal, we performed two additional dN/dS calculations. In the first, Gblocks was set to remove columns if they had one or more gaps. In the second, we calculated dN/dS based on the alignment not pruned by Gblocks.

### Analysis of AT Content, Codon Usage, and Amino Acid Frequencies

Codon usage and amino acid frequencies were calculated using CodonW 1.4.2 (Peden, 1999) based on the subset of protein-coding genes present in all completely assembled plastomes of *Thismia, B. disticha*, *B. coelestis*, *T. chantrieri*, and *T. leontopetaloides*. To compare amino acid frequencies between *Thismia* and photosynthetic species, we used the function OUWie from the package of the same name (Beaulieu et al., 2012). By fitting Ornstein-Uhlenbeck processes to the phylogeny, the function OUWie allows estimation of whether several clades have significantly different values of some parameter. For each amino acid, we tested the model by assuming a single Ornstein-Uhlenbeck process for the whole tree, or a model with two Ornstein-Uhlenbeck processes, where the “center parameter” of the model changes on the branch where photosynthesis is assumed to have been lost. *P*-values were calculated by the likelihood ratio test and then the Benjamini-Hochberg correction was performed for multiple tests. This allowed us to compare differences in usages of particular amino acids. In addition, we analyzed the tendency to use amino acids with more AT-rich codons. This “tendency” was calculated for each species as the Spearman’s correlation coefficient between the frequencies of amino acids in plastid-encoded proteins and the average AT contents of codons of these amino acids. The tendencies were compared between *Thismia* and photosynthetic species using OUWie, using the same method we used for frequencies of separate amino acids, except that the Benjamini-Hochberg correction was not needed as there was only a single test performed. To forecast the future evolution of AT content, we used the “equilibrium AT content,” the AT content that would established if the frequencies of nucleotide substitutions stay the same as at present. The equilibrium AT content can be considered an asymptote toward which the AT content of some sequence is currently heading.

If we denote guanine or cytosine as [GC] and adenine or thymine as [AT], then nucleotide substitutions can be represented as

(1) $$[\text{GC]}\begin{array}{c} {\text{k}}_{1}\\ ⇄\\ {\text{k}}_{2} \end{array}[\text{AT]}$$

where k_1_ is the frequency of substitution of [GC] by [AT] and k_2_ is the frequency of substitution of [AT] by [GC]. If we denote by N_*GC*_ the number of [GC] in a genomic sequence and by N_*AT*_ the number of [AT] in the genomic sequence, then the rate of change of N_*GC*_ is:

(2) $$\frac{{\text{dN}}_{\text{GC}}}{\text{dt}}=k_{2}\times N_{\text{AT}}-k_{1}\times N_{\text{GC}}$$

By N_*AT–eq*_ and N_*GC–eq*_ we can derive the numbers of [AT] and [GC] when an equilibrium is reached.

The equilibrium AT content is then:

(3) $$\alpha_{e\,q}=\frac{{\text{N}}_{\mathrm{AT}-\mathrm{eq}}}{\left(N_{\text{AT}\,\text{eq}}+N_{\text{GC}\,\mathrm{eq}}\right)}$$

When the equilibrium is reached, N_*GC*_ stops changing, and so:

(4) $$\frac{{\mathrm{dN}}_{\mathrm{GC}}}{d\,t}=0$$

From equations (2) and (3), it follows that:

(5) $$\alpha_{e\,q}=\frac{K\,1}{K\,1+K\,2}$$

The calculation of the equilibrium AT content was performed for the concatenated sequences of common protein-coding, rRNA-coding and tRNA-coding genes of *Thismia* and *D. elephantipes* after pruning by Gblocks. To calculate k_1_ and k_2_, which are the rates of [GC] → [AT] and [AT] → [GC] substitutions, we performed an ancestral sequence reconstruction by FastML 3.11 (Moshe and Pupko, 2019), separately for protein-coding genes, and for rRNA- and tRNA-coding genes.

GTR+Gamma models were used in both cases. Ancestral indels were reconstructed by the maximum likelihood method. Then the results of the reconstructions for these two gene sets were concatenated and the calculations described further were performed for the concatenated sequences. k_1_ for each branch was calculated as the number of [GC] → [AT] substitutions divided by N_GC_, in turn divided by the time that the branch existed. Similarly, k_2_ was calculated as the number of [AT] → [GC] substitutions divided by N_AT_ and then divided by the time that the branch existed. Because the time cancels out in equation (5), we did not need to calculate it. Confidence intervals for the equilibrium AT content were calculated by performing these analyses for 1000 bootstrap pseudoreplicates of the alignment, taking the 2.5th and the 97.5th percentiles of the equilibrium AT content.

### Other Analyses

To calculate mutation frequencies in different nucleotide contexts (i.e., the nucleotides that surround a given nucleotide) we used the ancestral reconstruction made by FastML for protein-coding genes. For each branch, we counted the number of synonymous substitutions that happened in a specific nucleotide context and the total number of synonymous substitutions that could happen in that context, including those that did not happen. For each context we summarized those values over all branches in the clade of *Thismia* and divided the first value by the second, thus obtaining the frequencies of substitutions in specific contexts. Assuming that synonymous substitutions are approximately neutral, this value can be used as a proxy for mutational frequencies in contexts.

## Results and Discussion

### Plastome Assembly, Structure, and Gene Content

**FIGURE 1:**
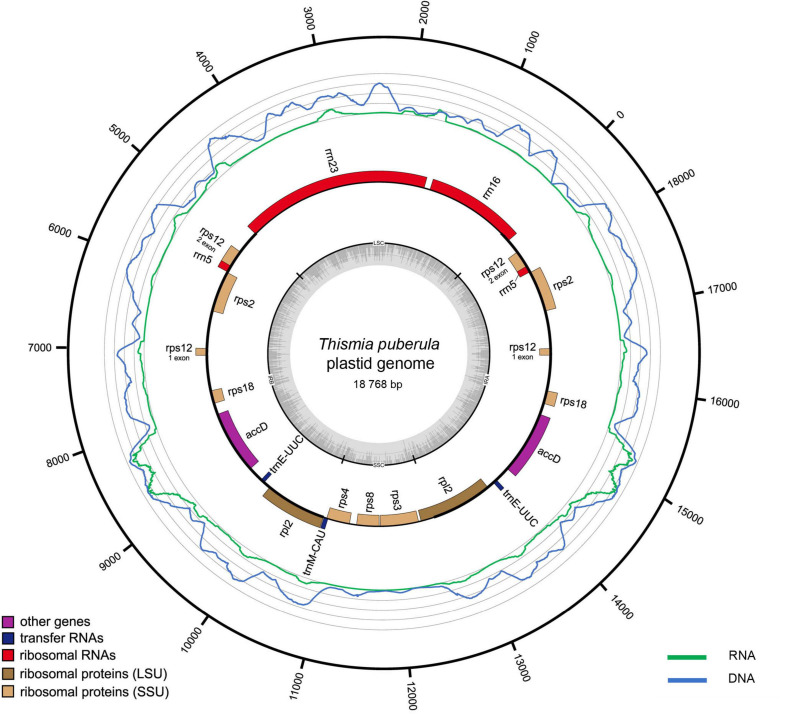
Plastid genome of *Thismia puberula*, its GC-content, gene expression and coverage by genomic DNA sequencing reads. This species has the largest gene set and was available as a living plant allowing to analyze the transcriptome. Dark gray dashes denote GC content; thick black lines denote IR and thinner lines denote single copy regions. Genes shown outside of the circle are transcribed counterclockwise and those inside are transcribed clockwise. Green and blue lines show coverage by transcriptome reads and genomic DNA reads. Gray circles correspond to a 1000x coverage for transcriptome and 2000x for DNA.

The number of reads obtained for each species varied from 1,312,880 to 40 million (Supplementary Table 1). For all species studied, at least one plastid contig was recovered. We assembled complete sequences for seven species, and partial sequences comprising from 8 to 14 genes for ten others (Figure 1 and Supplementary Figure 1). The inability to obtain complete sequences was not related to the low coverage. This may be related to factors that adversely affect the efficiency of the assembly, such as repeats or low-complexity regions, the presence of contaminants and/or mitochondrial DNA of plastid origin.

The length of complete plastomes ranged from 14,060 bp in *Thismia thaithongiana* Chantanaorr. & Suddee to 18,768 bp in *T. puberula* (Table 1). They all have a typical quadripartite structure including large single copy (LSC) and small single copy (SSC) regions divided by an inverted repeat (IR) region. As expected, the gene set in these full mycoheterotrophs was drastically lower compared with that in photosynthetic plants. For the majority of studied species, we found a large IR that includes *accD*, *rps2*, *rps12*, *rps18*, *rpl2*, *rrn5*, *trnE-UUC*, and *trnfM-CAU*. The LSC typically consists of *rrn16* and *rrn23*, and the SSC of *rps3*, *rps4*, and *rps8*. The IR of *T. tentaculata* was much shorter than others and included only *trnM*, *rpl2*, *trnE*, and *accD* (Lim et al., 2016). On a smaller scale, there were minor variations in the position of IR-SC borders. At the IR-SSC border, the IR included *trnfM-CAU* and/or the second exon of *rpl2* in *Thismia filiformis* Chantanaorr., *Thismia gardneriana* Hook.f. ex Thwaites, *T. tentaculata*, *T. thaithongiana* and lacked these genes in the other species. At the IR-LSC border, a small contraction of IR was observed in *T. thaithongiana* where it ends in *rps12* instead of the more usual *rrn16*.

**TABLE 1:** Characteristics of complete plastomes of *Thismia*.

**Species**	**Total length, bp**	**LSC, bp**	**SSC, bp**	**IR, bp**	**GC- content, %**
*Thismia filiformis*	17480	4439	1682	5680	26.72
*Thismia gardneriana*	17645	4584	1750	5655	27.37
*Thismia hawkesii*	17312	4675	1796	5421	25.70
*Thismia lanternata*	18428	4822	1875	5865	27.09
*Thismia mucronata*	18718	4673	1960	6042	26.67
*Thismia puberula*	18768	4733	1952	6042	26.65
*Thismia thaithongiana*	14060	5105	1252	3851	20.07

Five genes – *accD*, *rpl2*, *rrn16*, *rrn23*, *trnE* – were present as complete genes in all complete plastome sequences; for the first two genes, an open reading frame (ORF) with a typical start and stop and a domain characteristic for a given protein was found. We also found all five genes, at least as a fragment, in the uncompleted genomes; thus, these genes likely constitute a core set of plastid genes in *Thismia* (Supplementary Table 3).

The plastomes of most species also had *rps2*, *rps12*, *rps4*, *rps8*, *rps18*, *rrn5*, *trnM*, and *rps3*. In most cases, these genes have a high similarity to orthologs from photosynthetic species and a typical structure (ATG start codon and ORF length in the protein-coding genes), with some deviations. In *T. thaithongiana*, all genes from SSC (all protein-coding) lack an ATG start codon. Also, *rps4* and *rps8* were ∼100 bp shorter in this species than in the other studied species; however, both genes retained at least a part of the domain typical for these proteins (Supplementary Table 4). Although the emergence of a start codon not homologous with the ancestral one is known to occur in plastid genes (Barthet et al., 2015), *rps3*, *rps4*, and *rps8* in *T. thaithongiana* lack any in-frame ATG (or ACG which could be converted to AUG by RNA editing) that could serve as an alternative start of translation near the 5′-end of the ORF. This suggests that they are pseudogenes in this species. In *T. gardneriana*, *rps3* and *rps8* also lack a typical start codon; the only in-frame ATG in their ORF is located near the end and is unlikely to act as an alternative start for translation. Both *rps3* and *rps8* proteins in this species are highly similar to the orthologs from the other species studied here and retain the corresponding domains. The same is true for *rps4* in *T. filiformis* and *T. lanternata*. In *T. tentaculata*, *rps3* also lacks an ATG start codon, and a C-terminal S3 domain is not present. We found an ORF with similarity to *rps3* in all complete plastomes; in three species (*T. lanternata*, *T. mucronata*, and *T. puberula*) we did not detect its S3 domain in the corresponding proteins despite the ORFs having a typical start.

The *rps2* gene is missing in two species: *T. hawkesii* and *T. thaithongiana.* In *Thismia hongkongensis* Mar & R.M.K.Saunders species *rps2* is likely pseudogenized: we found a region with high (92%) similarity to *rps2* of the sister species, *T. tentaculata*, but it carries two stop codons in the middle of the gene.

The *rps18* gene is completely missing in *T. filiformis*. This is the only complete plastome where we did not detect *rps18*; however, the examination of partial sequences in the region typically containing *rps18* (i.e., between *rps12* and *accD*) showed that in *Thismia alba* Holttum ex Jonker and *Thismia hexagona* Dančák, Hroneš, Kobrlová & Sochor this region also lacks *rps18*, and in *Thismia neptunis* Becc. it has a probable *rps18* pseudogene. Given that all the studied *Thismia* plastomes are collinear (except for the variation of the IR borders), it is reasonable to predict that *rps18* has indeed been lost in *T. alba*, *T. hexagona*, and *T. neptunis* and not translocated to another region which possibly was not assembled. Following the same argumentation, we predict *rps8* to be missing in *Thismia kelabitiana*, Dančák, Hroneš & Sochor, because the partial assembly of its plastome contains *rps3* and *rps4*, but lacks *rps8* which is arranged between the two former genes in the other *Thismia* species studied here.

The plastid genes exhibit extensive length variation in *Thismia*. For instance, in *T. thaithongiana* several genes (*rps4*, *rps18*, *rrn23*) are shorter than in other species, and in *T. hongkongensis* we observed an increase of *accD* length compared to another *Thismia* species from approximately 1100 to 1422 bp. *rpl2* has an unusual start codon – ACG – in all species examined. For *T. puberula*, we experimentally confirmed that RNA editing converts the ACG start codon to AUG (see below).

### Evolution of Plastid Genes

The plastid genes in *Thismia* have a high level of sequence divergence compared to related photosynthetic taxa. The most conserved regions are tRNA and rRNA genes and the most variable ones are *rps3* and *rps4* (Supplementary Table 5). The dN/dS value varied from 0.25 to 0.45 (Table 2); notably, the lower limit is close to the dN/dS values found in other non-photosynthetic plants with a highly reduced plastome (Lam et al., 2015; Schelkunov et al., 2015), whereas the higher values may indicate relaxed purifying selection. We found a significant difference in dN/dS of plastid genes of *Thismia* and their orthologs in photosynthetic plants for most genes, except for *rps8* and *rpl2* (Table 2). These results should be treated with caution, however, as dN/dS analysis is highly sensitive to the alignment quality and in *Thismia*, due to their rapid evolution, biased nucleotide content and high frequency of indels, small misalignments are more probable. The same is true for other plants with highly reduced plastomes; for this reason Peterson and co-workers decided not to perform selection pressure analysis for *Sciaphila* (Petersen et al., 2018). Taking into account the uncertain status of *rps3*, *rps4*, and *rps8* in several species where we were not able to find the start codons in these genes (see above), we performed dN/dS analysis for these genes in two variants: including and excluding the sequences with an atypical predicted start codon. The exclusion of these sequences did not result in lower dN/dS values (Table 2). We consider this to be a result of greater variability of genes lacking typical start codon; their inclusion in the alignment leads to the removal of a higher number of low-conserved regions by Gblocks. dN/dS calculations for multiple alignments not pruned by Gblocks (Supplementary Table 6) or pruned by Gblocks with aggressive gap-removal behavior (Supplementary Table 7) indicate that the gap-removal parameters of Gblocks has a minor effect on the dN/dS estimates.

**TABLE 2:** dN/dS values for species of *Thismia* and their photosynthetic relatives and *p*-value and *q*-value for the hypothesis of the difference of dN/dS values between photosynthetic and non-photosynthetic species.

**Gene**	**dN/dS in *Thismia* species**	**dN/dS in photosynthetic plants**	***p*-value**	***q*-value**
*accD*	0.41	0.29	0.00	0.01
*rpl2*	0.32	0.23	0.59	0.59
*rps2*	0.43	0.13	8.26 × 10^−8^	4.54 × 10^−7^
*rps3*	0.35	0.12	1.09 × 10^−6^	2.40 × 10^−6^
*rps3*, with start sequences atypical codon excluded	0.47	0.12	4.45 × 10^−7^	1.22 × 10^−6^
*rps4*	0.36	0.1	2.86 × 10^−7^	1.05 × 10^−6^
*rps4*, sequences with atypical start codon excluded	0.37	0.1	6.80 × 10^−8^	7.48 × 10^−7^
*rps8*	0.23	0.26	0.49	0.54
*rps8*, sequences with atypical start codon excluded	0.26	0.26	0.24	0.30
*rps12*	0.25	0.03	0.00	0.00
*rps18*	0.44	0.31	0.00	0.00

**FIGURE 2:**
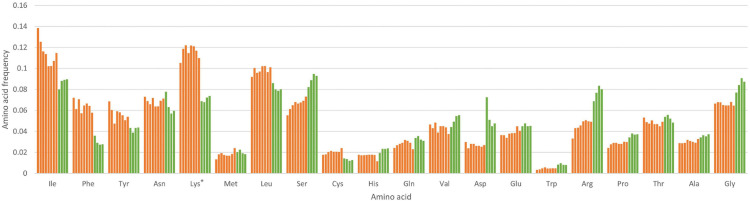
Amino acid content of plastid genes in *Thismia* and photosynthetic Dioscoreales. Values for *Thismia* are depicted by orange bars, values for photosynthetic species by green bars. The species, from left to right: *Thismia thaithongiana*, *Thismia hawkesii*, *Thismia lanternata*, *Thismia filiformis*, *Thismia mucronata*, *Thismia puberula*, *Thismia gardneriana*, *Thismia tentaculata*, *Burmannia coelestis*, *Burmannia disticha*, *Tacca chantrieri*, *Tacca leontopetaloides*. Asterisk indicates statistically significant difference between *Thismia* species and photosynthetic species (*q*-value < 0.05).

A prominent feature of *Thismia* plastomes is their high AT-content compared to photosynthetic plants. Generally, increased AT-content (in relation to that of autotrophic plants) is typical for plastomes of non-photosynthetic plants, and the level of AT-richness correlates with the degree of plastome reduction. In species with a recent transition to full heterotrophy, AT-bias is exhibited only at the level of synonymous codon usage; in highly reduced plastomes it affects amino acid content through a high amount of amino acids coded by the AT-rich codons (Lys, Phe) (see, e.g., Schelkunov et al., 2015). The latter tendency is also observed in *Thismia* (Figure 2), although when counted at the level of individual amino acids it is statistically significant only for lysine. The correlation between the frequency of an amino acid and the (average) AT-richness of a codon that encodes this amino acid is significantly higher in *Thismia* than in the autotrophic species of *Tacca* and *Burmannia* (belonging to the same order Dioscoreales) (Supplementary Table 8). Also, our analysis shows that the correlation values differ greatly between some photosynthetic plants, even between closely related taxa such as *Burmannia coelestis* and *Burmannia disticha*. This indicates that the bias toward AT-richness could predate the transition to heterotrophy, at least in some cases. In order to gain some insight into the evolutionary trends of nucleotide content in plastid genes of *Thismia*, reveal its ancestral state, and to make a prognosis for the future state we performed an analysis of the equilibrium AT content. In brief, this is the AT-content that a genomic sequence will reach if the [GC]- > [AT] substitutions and [AT]- > [GC] substitutions retain their current frequencies. In other words, the equilibrium AT content is the asymptote to which the AT content of a sequence is currently heading. The analysis of the concatenated gene sequence of each of the completely assembled *Thismia* plastomes (Supplementary Figure 2) indicated that in at least five out of nine species the AT content has not reached its peak yet, but still continues to increase. A notable case is the plastome of *Thismia thaithongiana*, for which the equilibrium AT-content is approximately 92%. AT-content of plastid genes more than 90% is known for Balanophoraceae (Schelkunov et al., 2019; Su et al., 2019; Chen et al., 2020), therefore it may be possible to actually achieve such a high AT-content. However, mutation spectra and selection in *Thismia* may change in the future thus making the current equilibrium AT-contents different from stable AT-contents that *Thismia* will finally reach.

The analysis of substitution spectra and their contexts can give insights into the possible causes that underlie changes in the genomic sequence – for example, environmental factors such as UV light, or intrinsic genome properties such as methylation (Ossowski et al., 2010; Kusmartsev et al., 2020). We analyzed the frequencies of synonymous substitutions in different contexts in the plastid genes of *Thismia* species. The result (Supplementary Figure 3) shows that the main type of substitution in the genus *Thismia* is the C- > T transition (equivalent to the G- > A transition on the complementary strand) without a distinct context.

### Transcriptome Data

The survey of expression of plastid genes in *T. puberula* showed variation in gene expression (Supplementary Table 9, column C). It should be noted that for genes coding for ribosomal RNA, the expression levels are presumably artificially lowered because we used a ribosomal RNA depletion kit for library preparation, which includes probes for plastid rRNAs. The expression level is the highest in *accD*, the only gene that is not related to the translation apparatus (all other retained genes are for this apparatus). We did not detect the expression of genes from the SSC (*rps3*, *rps4*, and *rps8*); however, this may be because these genes have the highest AT-content (92% in *rps3*, 84% in *rps4*, and 86% in *rps8*) and highly AT-rich regions are known to be problematic for Illumina sequencing (Oyola et al., 2012).

The *rpl2* gene has an atypical start codon that is hypothesized to be converted into a typical one by RNA editing. Indeed, we detected the editing, but at a low level: only ∼20% of reads contained the edited cytosine (Supplementary Figure 4).

The genes *rps12* and *rpl2* are annotated as intron-containing. For *rpl2*, we could not unambiguously detect evidence of splicing, but the coverage of RNA-seq reads in the intron region is lower than in exons (Figure 1). *rps12* has an unusual genomic location in *Thismia*: its second exon precedes the first one. In photosynthetic plants, *rps12* is known to be transcribed as two separate transcripts, one containing the first exon and the other containing the second and the third exons; the three exons are then *trans-*spliced (Hildebrand et al., 1988, p.12). We assume that the exons of *rps12* are also transcribed separately in *Thismia*. Generally, many plastid genes are known to be transcribed as polycistronic transcripts in plants. Thus we performed *de novo* assembly of the transcriptome data in order to find co-expressed genes. Overall, 11 contigs were found (Supplementary Table 7). The longest one is ∼2.8 Kb and contains complete sequences of *rps18*, *accD*, and *trnE* indicating that these genes are co-transcribed.

### Phylogenetic Analysis

**FIGURE 3:**
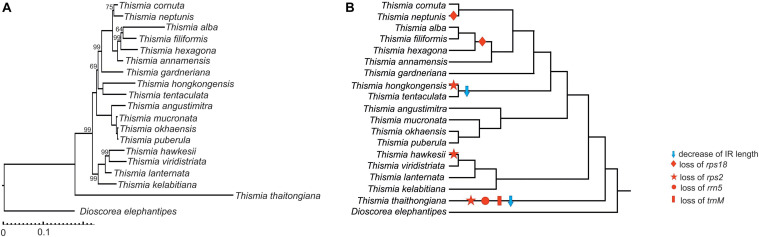
Phylogenetic tree based on *Thismia* plastid genes. **(A)** Phylogenetic tree inferred from ML analysis of 13 plastid genes by IQ-Tree with branch lengths and bootstrap values (only values < 100 are shown). **(B)** The same tree showing gene losses and IR borders changes.

All three analyses recovered the same tree topologies, with generally strong branch support from bootstrap analyses or posterior probabilities for individual branches (Figure 3 and Supplementary Figure 5). This topology is similar to one inferred in an earlier report based on the analysis of nuclear (18S, ITS) and mitochondrial (*atp1*) sequences in a larger set of species (Shepeleva et al., 2020), which revealed five major clades that are also consistent with morphological and geographical evidence. Our dataset includes the representatives of four of these clades, and all four clades are recovered with high support in our reconstructions. The only differences are the position of *T. hongkongensis*, which was inferred to be the sister group of *T. gardneriana* in the nuclear/mitochondrial tree (Shepeleva et al., 2020) and to *T. tentaculata* in the plastid tree (Figure 3), and the relative positions of *T. alba*, *T. hexagona*, and *T. filiformis*. However, these relationships have low support in the nuclear/mitochondrial tree, likely indicating a lack of variable positions or conflicting phylogenetic signal in these datasets. The reconstruction of evolutionary events leading to the observed pattern of gene losses supports three independent losses of *rps2* and two losses of *rps18* (Figure 3B).

### Plastome Size and Structure in *Thismia* Compared to Other Heterotrophic Plants

Our study shows that the plastomes of *Thismia* are at the latest stages of genome reduction. They follow the general patterns found in the plastomes of other non-photosynthetic plants: the loss of genes, nucleotide bias toward AT, and protein sequence bias toward amino acids coded by AT-rich codons (Schelkunov et al., 2015, 2019). Notably, the plastomes of *Thismia* have almost the same length as those of Balanophoraceae (Schelkunov et al., 2019; Su et al., 2019) and *Epipogium roseum* Lindl. (Orchidaceae) (Schelkunov et al., 2015) and are larger than the plastome of *Sciaphila thaidanica* K.Larsen (Triuridaceae) (Petersen et al., 2018), whereas the gene content in plastomes of *Thismia* is more reduced. In contrast to *Thismia*, Balanophoraceae retain *ycf1* and *ycf2* (two large genes encoding proteins required for protein import into plastids), *clpP*, and several ribosomal protein genes. *Epipogium roseum* lacks *ycf1* and *ycf2* but retains *clpP* and a larger set of ribosomal protein genes. The 12-kb plastome of *S. thaidanica* lacks *ycf* and *clpP* but retains five additional ribosomal protein genes compared to *Thismia*. The large size of *Thismia* plastomes (in relation to the gene set) is mostly a result of the large inverted repeat (IR). In their investigation of *T. tentaculata*, Lim et al. (2016) suggested that the IR in *Thismia* plastomes could have been acquired secondarily after a complete loss in a common ancestor of the genus. Such re-emergence of an IR was recently shown in Fabaceae, in a clade where the absence of IR is the ancestral state (Choi et al., 2019). Our study, based on an expanded species sampling in the genus revealed that the IR position in most species of *Thismia* differs from that of *T. tentaculata*: it generally includes *rps18*, *rps12* (both exons), *rps2*, and *rrn5* (in the species where this gene is present). We found this IR structure in most *Thismia* species including the sister group of other members of the genus, *T. thaithongiana*. This implies that it represents the ancestral state for the Old World clade of *Thismia*. *rps12* (3′-end) and *rrn5* are a part of typical IR in flowering plants. We thus suggest that the unusual IR of *Thismia* arose not completely *de novo* but rather as a combination of a small part of typical IR (3′-end of *rps12* and *rrn5*) and a part of LSC adjacent to IRb (*accD*, *rps18*, 5′-end of *rps12*). We hypothesize that the SSC of *Thismia* (consisting of *rps4*, *rps8*, and *rps3*) arose from the ancestral LSC, and the LSC from a part of the typical IR. A survey of plastome structure in the genera of Thismiaceae phylogenetically close to *Thismia* (*Tiputinia*, *Oxygyne*, and *Haplothismia*) and the New World clade of *Thismia* would help to outline this path in more detail.

### Plastome Diversity and Evolution in *Thismia*

The loss of plastid genes caused by the transition to heterotrophy is presumably irreversible (e.g., Barrett and Davis, 2012; Graham et al., 2017). While it is possible that genes integrated in the nuclear or mitochondrial genomes (indeed, transfers from the plastid to the nuclear and mitochondrial genome are frequent in plants, see, e.g., Martin and Herrmann, 1998; Wang et al., 2007) before being lost from the plastome can hypothetically be transferred back, no such cases have been observed up to date. Our recent study of the mitochondrial genome of another mycoheterotropic plant with a highly reduced plastome, *Hypopitys monotropa* Crantz, showed that while it carries the fragments of plastid genes that are absent in the present-day plastome, none of them retains potentially functional ORFs (Shtratnikova et al., 2020). Also, an investigation of the transcriptomes of three mycoheterotrophic species did not reveal that any genes lost from the plastome were expressed elsewhere (Schelkunov et al., 2018). Indeed, experiments show that the genes transferred from the plastome to the nuclear genome are rapidly lost (Sheppard and Timmis, 2009). Therefore, we consider gene loss in heterotrophic plants to be an irreversible process. As shown earlier (Lim et al., 2016), in *Thismia tentaculata* the genes responsible for photosynthesis-related processes as well as many ribosomal protein and tRNA genes are lost. The examination of a diversity of gene sets in the plastomes of other *Thismia* species reveals a tendency to further reduction. At least five genes (*trnM*, *rrn5*, *rps2*, *rps18*, and *rps3*) found in the genus are lost (either completely or pseudogenized) in one or more species. The mapping of gene losses on the phylogenetic reconstruction shows that *rps2* and *rps18* were lost independently in different clades (Figure 3B). In particular, *rps18* was lost in a common ancestor of *T*. *alba*, *T. hexagona* and *T. filiformis* and in *T. neptunis*. *rps2* was lost in *T. thaithongiana*, *T. hawkesii* and *T. hongkongensis*. Of these, *T. thaithongiana* presumably possesses the most ancient loss because this species lacks any sequences with significant similarity to *rps2*, whereas the two other species carry putative pseudogenes of *rps2*. Although *T. thaithongiana* is the only species from the clade 1 (recognized by Shepeleva et al., 2020) sampled in our dataset, the situation observed in clade 5, where the loss of *rps18* is a hallmark of closely related species, allows us to speculate that the loss of *rps2* is not unique for *T. thaithongiana* but shared by a group species from clade 1.

The modifications occurring in *Thismia* plastomes are not limited to gene losses. The length of some genes also varies greatly within the genus. Thus, in *T. thaithongiana*, several genes (*rps8*, *rrn23*, *rps4*) are 20–40% shorter than the average for the genus. In *T. hongkongensis* we observed an unusual structure of the *accD* gene: its 5′-end codes for a ND–rich sequence, which lacks significant similarity either to *accD* or to any other known protein. Reconfigurations of *accD* and, in particular, large insertions are known in a number of photosynthetic (Park et al., 2018; Sudianto and Chaw, 2019) and non-photosynthetic (Logacheva et al., 2016) species. However, in the previously known cases they do not affect the carboxylase domain, the only known functional domain of *accD* that lies in the C-terminus. The same is true for *T. hongkongensis*: the 3′-end of *accD* shows high similarity with other species while its 5′-end is modified.

The retention of *accD* in all studied species of *Thismia* is congruent with its function (synthesis of fatty acids) which is not related to photosynthesis. The presence of this gene is typical for non-photosynthetic plants, even for those with plastomes more reduced than those of *Thismia* (Bellot and Renner, 2016; Graham et al., 2017; Arias-Agudelo et al., 2019). On the other hand, the plastid-encoded *accD* is missing in several autotrophic plants, being replaced by its nuclear plastid-targeted counterpart (Rousseau-Gueutin et al., 2013). This example emphasizes that despite some general patterns of gene loss present in heterotrophic plants, the necessity of a certain gene can be lineage-specific, as we noted earlier (Schelkunov et al., 2015). In line with this idea, plastomes of *Thismia* bear an intact *trnE* gene, seeming to support the essential tRNA hypothesis (Barbrook et al., 2006), in line with nearly all heterotrophic plants (e.g., Graham et al., 2017), whereas it appears to be absent in *Pilostyles* (Apodanthaceae) (Bellot and Renner, 2016). The survey of the transcriptome of *Thismia puberula* supports the importance of *accD*, revealing its expression level to be the highest among plastid genes. Also, the transcriptome data indicate that the set of functional genes encoded in the *Thismia* plastomes is possibly even smaller than annotated: all genes from the SSC (*rps3*, *rps4*, and *rps8*) are not expressed (it should be noted, however, that the AT content of these genes is the highest among all protein-coding genes of *T. puberula* and this can lead to artificial underestimation of expression levels). Together with the fact that these genes lack typical start codons in some species, this suggests that we possibly observed the first step toward pseudogenization of these genes in *Thismia*.

### Employment of Plastid Markers in Phylogenetic Reconstructions

An important implication of our study is the finding that the plastomes of *Thismia* species, despite their unusual structure and reduced gene content, are valuable phylogenetic markers in *Thismia*. Plastid genes, and especially *rbcL* and *matK*, are extensively used for inferring plant phylogeny. Massive gene loss and biased nucleotide content in plastomes hamper their employment for the integration of heterotrophic plants in the plant tree-of-life. Surprisingly, the phylogenetic trees of *Thismia* inferred here from the plastid genes are similar to those inferred from nuclear and mitochondrial regions and provide higher resolution and support than any of the non-plastid trees. Our results are in agreement with the conclusions of Lam and co-workers who pointed out (Lam et al., 2016) that several plastid genes can be used for phylogenetic analysis in mycoheterotrophic plants. In particular, these authors suggested using *accD*, *clpP*, and *matK*, and reported that they could not find *clpP* and *matK* in Thismiaceae. Based on our data, we propose an addition of *rpl2* and *rps12* to this prospective gene set for phylogenetic inference.

The increasing availability of complete plastomes of Dioscoreales and especially Burmanniaceae (Ma et al., 2018; Li et al., 2019) also gives an opportunity to use larger datasets comprising the entire plastomes to reconstruct the relationships within this order and, in particular, to elucidate the phylogenetic vicinity of Thismiaceae (though this requires caution about long branch attraction – see Lam et al., 2018; Schelkunov et al., 2019). *Thismia* is a highly underexplored genus; more than 30 species of this genus were described after 2015 (e.g., Hroneš et al., 2018; Da Silva et al., 2020; Dančák et al., 2020b; Xu et al., 2020) and species complexes with uncertain boundaries between taxa become evident with discovery of new populations (Dančák et al., 2020b; Nuraliev et al., 2020). The majority of species of *Thismia* were described based exclusively on morphology, and the position of many of them in *Thismia* phylogeny remains unclear (Shepeleva et al., 2020). For some species already included in phylogenetic analyses, the nuclear 18S and mitochondrial *atp1* markers suggest ambiguous and controversial placement because of insufficient resolution. We expect that the plastid genes will help to resolve such cases due to their extremely high variability. This will be especially important for testing the identity and monophyly of species that are closely related and morphologically similar.

## Conclusion

We characterized plastid genomes in the genus *Thismia*, a group with an ancient transition to heterotrophy, based on a sampling of 18 species. As expected, plastomes of *Thismia* are at the late stages of the reduction; their gene set is smaller than in the plastomes of similar length in other groups of heterotrophic plants. The dense taxonomic coverage that includes species from four out of five major lineages of the Old World *Thismia* clade allowed us to outline the evolutionary patterns of plastome reduction and to predict further trends, which include further increase of the AT-content, the pseudogenization and loss of several ribosomal protein genes (such as all genes from the SSC). Our remarkable finding is that the phylogenetic trees inferred from the plastid genes of *Thismia* are similar to the trees inferred from nuclear and mitochondrial sequences. This supports the idea that the plastome sequences are valuable markers for further phylogenetic analysis of this large and diverse genus.

## Data Availability Statement

Nucleotide sequences generated in this study can be found in the online public database GenBank (https://www.ncbi.nlm.nih.gov/genbank/) under accession numbers MT943132, MT943130, MT943129, MT943131, MT936983, MT943140, MT936984, MT943134, MT943133, MT936982, MT936985, MT943141, MT943135, MT943136, MT943137, MT943139, MT747830, MT943142, and MT943138.

## Author Contributions

SY performed assembly and annotation and collected plant material. MIS performed the evolutionary and phylogenetic analysis and revised the manuscript. LN and DC sequenced and assembled plastomes of *T. hawkesii* and *T. lanternata*. SC, MH, MSo, MD, S-SM, and HTL collected the material. MN collected the material and revised the manuscript. ML conceived and coordinated the study, performed library preparation and sequencing, and drafted the manuscript. All authors contributed to the article and approved the submitted version.

## Conflict of Interest

The authors declare that the research was conducted in the absence of any commercial or financial relationships that could be construed as a potential conflict of interest.

## Footnotes

1 https://www.geneious.com

2 https://github.com/JGI-Bioinformatics/Kmernator

## Supplementary Material

The **Supplementary Tables 1–9** can be found in the Table 1 file.

Supplementary Figure 1 Maps of complete and partial plastomes of *Thismia* reported in this study and the morphology of the species for which sequences were obtained in this study. **(A)** *T. alba* (Thailand). **(B)** *T. angustimitra* (Thailand). **(C)** *T. annamensis* (Nuraliev 999). **(D)** *T. cornuta* (Sochor et al. BOR24/17). **(E)** *T. filiformis* (Chantanaorrapint & Promma 3928). **(F)** *T. gardneriana* (Chantanaorrapint 2309). **(G)** *T. hawkesii* (Cooper 2407). **(H)** *T. hexagona* (Dančák et al. 2013/17). **(I)** *T. hongkongensis* (Hong Kong). **(J)** *T. kelabitiana* (Sochor et al. BOR1/17). **(K)** *T. lanternata* (Cooper 2403). **(L)** *T. mucronata* (Nuraliev 813). **(M)** *T. neptunis* (Sochor et al. BOR51/17). **(N)** *T. okhaensis* (Tich et al. KH 638B). **(O)** *T. puberula* (Yudina & Nuraliev 15). **(P)** *T. thaithongiana* (Chantanaorrapint 2755). **(Q)** *T. viridistriata* (Sochor et al. BOR06/19). Photographs: S. Chantanaorrapint **(A,E,F,P)**; M. Hroneš **(H)**; H.T. Luu **(N)**; S.S. Mar **(I)**; M.S. Nuraliev **(C,L,O)**; M. Sochor **(D,J,M,Q)**; P. Tripetch **(B)**; reproduced with permission from Austrobaileya 10: 133, 135 (2017) **(G,K)**.

Supplementary Figure 2 Evolution of AT content in plastid genes of *Thismia*. Blue numbers denote AT contents in current and ancestral sequences. Red numbers denote equilibrium AT contents with their 95% confidence intervals (CIs). Asterisks indicate the species for which the lower bound of the confidence interval for the equilibrium AT content exceeds the current AT content.

Supplementary Figure 3 Synonymous substitution profile in plastid genes of *Thismia*. Horizontal axis – synonymous substitutions with their sequence contexts (i.e., adjacent bases). Vertical axis – substitution frequencies. Four synonymous substitutions that are prohibited by the genetic code are marked by gray crosses. For complementary pairs of substitutions, like ACG- > AGG and CGT- > CCT, the one with C or T in the middle is shown.

Supplementary Figure 4 Mapping of RNA-seq on *T. puberula* plastome, the region of *rpl2*. Note the polymorphism at the second nucleotide of *rpl2* CDS due to RNA editing.

Supplementary Figure 5 Phylogenetic trees based on plastid genes of *Thismia*, **(A)** Bayesian (MrBayes) and **(B)** maximum likelihood (RAxML) analyses. Only posterior probabilities less than 1 and bootstrap support values less than 100 are shown.

Supplementary Table 1 Material used in this study, sequencing settings and accession numbers of plastome sequences.

Supplementary Table 2 Primers used for the check of IR-SC junctions in *T. mucronata*.

Supplementary Table 3 Genes found in plastomes of *Thismia*. “Gene absent” denotes cases where gene is either not found in the complete sequence or in a segment of partial sequence where it could have been located assuming the co-linearity of *Thismia* plastomes. “Gene not found” denotes cases where the whole segment is not found in the assembly. “Fragmented” denotes cases where a partial sequence of a gene is present in the assembly on a contig border.

Supplementary Table 4 Results of Pfam-a search for plastid genes of *Thismia*.

Supplementary Table 5 Sequence similarity of plastid genes of *Thismia*.

Supplementary Table 6 Estimates of selective pressure in plastid genes of *Thismia* and their photosynthetic relatives calculated using a multiple alignment not pruned by Gblocks. *P*-values and *q*-values indicate statistical significance of the hypothesis of the difference of dN/dS values between photosynthetic and non-photosynthetic species.

Supplementary Table 7 Estimates of selective pressure in Thismia and their photosynthetic relatives calculated using a multiple alignment pruned by Gblocks with default parameters. *P*-values and *q*-values indicate statistical significance of the hypothesis of the difference of dN/dS values between photosynthetic and non-photosynthetic species.

Supplementary Table 8 Spearman correlation between AT-richness of a codon (average in case of amino acids coded by multiple codons) and a frequency of amino acid.

Supplementary Table 9 Transcriptome contigs of *Thismia puberula* corresponding to plastid genome.
