# Supplementary figures and images for: Comparative Analysis of Plastid Genomes in the Non-photosynthetic Genus Thismia Reveals Ongoing Gene Set Reduction
Source: Front Plant Sci. 2021 Mar 16;12:602598. doi: 10.3389/fpls.2021.602598 (PMC8009136; doi:10.3389/fpls.2021.602598)

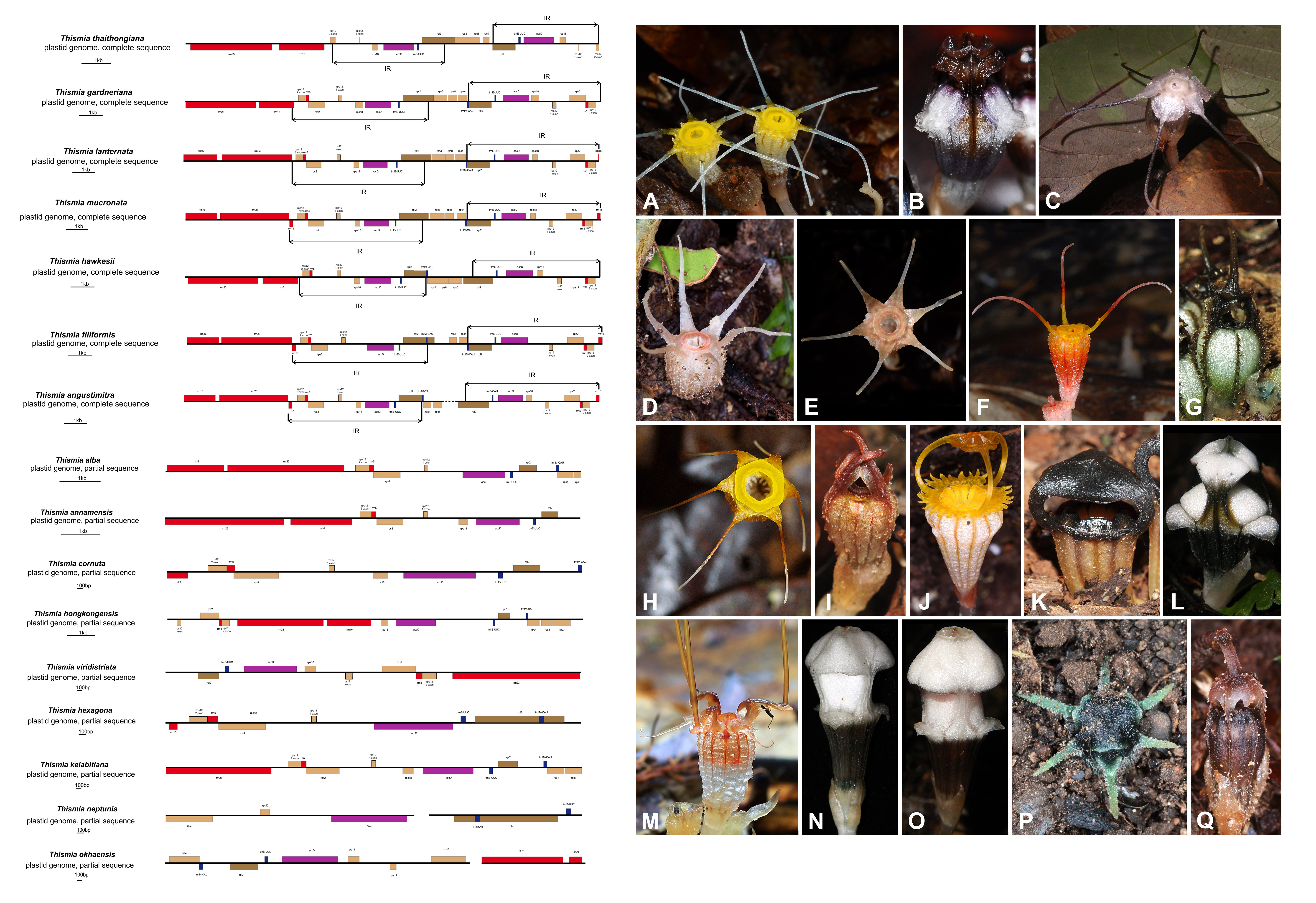

Supplement: Supplementary Figure 1 — Maps of complete and partial plastomes of Thismia reported in this study and the morphology of the species for which sequences were obtained in this study. (A) T. alba (Thailand). (B) T. angustimitra (Thailand). (C) T. annamensis (Nuraliev 999). (D) T. cornuta (Sochor et al. BOR24/17). (E) T. filiformis (Chantanaorrapint & Promma 3928). (F) T. gardneriana (Chantanaorrapint 2309). (G) T. hawkesii (Cooper 2407). (H) T. hexagona (Dančák et al. 2013/17). (I) T. hongkongensis (Hong Kong). (J) T. kelabitiana (Sochor et al. BOR1/17). (K) T. lanternata (Cooper 2403). (L) T. mucronata (Nuraliev 813). (M) T. neptunis (Sochor et al. BOR51/17). (N) T. okhaensis (Tich et al. KH 638B). (O) T. puberula (Yudina & Nuraliev 15). (P) T. thaithongiana (Chantanaorrapint 2755). (Q) T. viridistriata (Sochor et al. BOR06/19). Photographs: S. Chantanaorrapint (A,E,F,P); M. Hroneš (H); H.T. Luu (N); S.S. Mar (I); M.S. Nuraliev (C,L,O); M. Sochor (D,J,M,Q); P. Tripetch (B); reproduced with permission from Austrobaileya 10: 133, 135 (2017) (G,K). [file Image_1.jpeg]

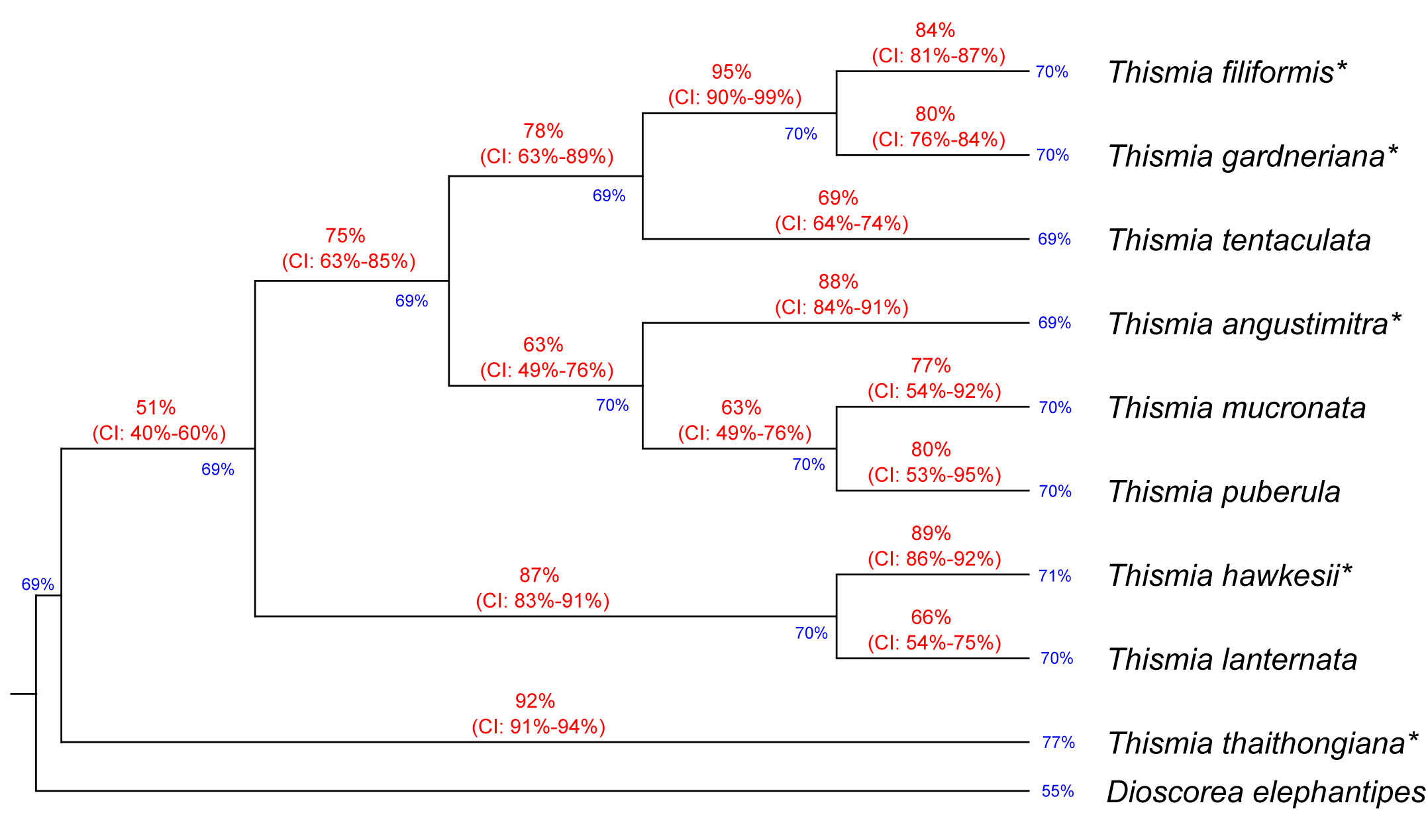

Supplement: Supplementary Figure 2 — Evolution of AT content in plastid genes of Thismia. Blue numbers denote AT contents in current and ancestral sequences. Red numbers denote equilibrium AT contents with their 95% confidence intervals (CIs). Asterisks indicate the species for which the lower bound of the confidence interval for the equilibrium AT content exceeds the current AT content. [file Image_2.jpeg]

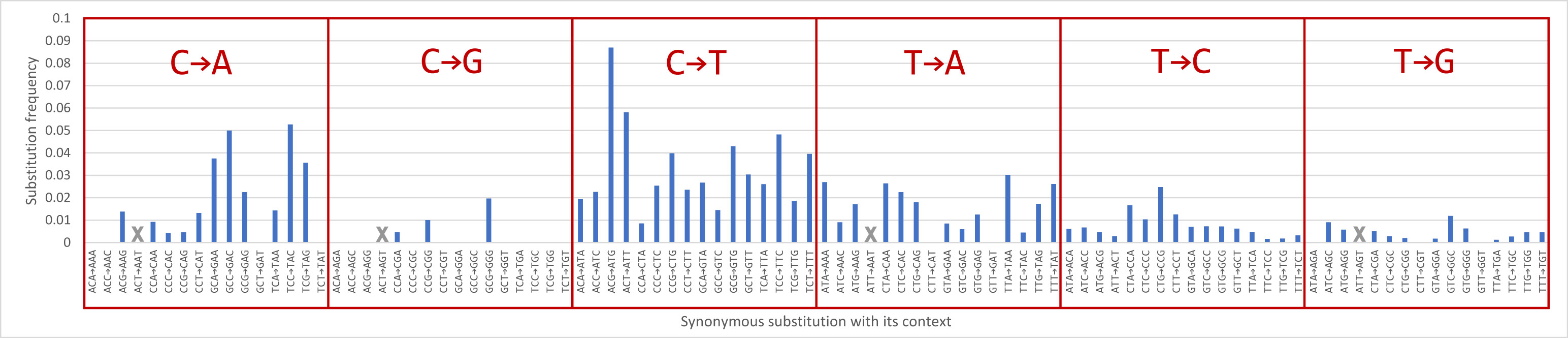

Supplement: Supplementary Figure 3 — Synonymous substitution profile in plastid genes of Thismia. Horizontal axis – synonymous substitutions with their sequence contexts (i.e., adjacent bases). Vertical axis – substitution frequencies. Four synonymous substitutions that are prohibited by the genetic code are marked by gray crosses. For complementary pairs of substitutions, like ACG- > AGG and CGT- > CCT, the one with C or T in the middle is shown. [file Image_3.jpeg]

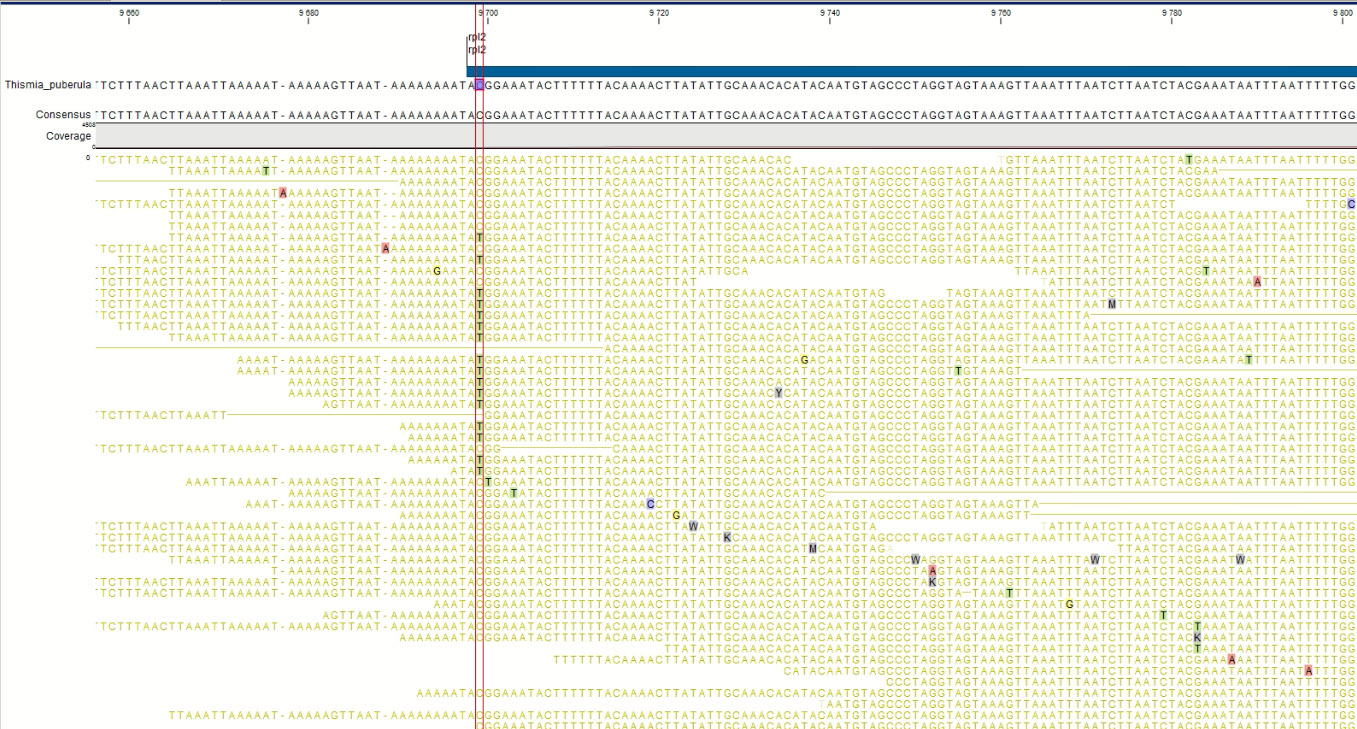

Supplement: Supplementary Figure 4 — Mapping of RNA-seq on T. puberula plastome, the region of rpl2. Note the polymorphism at the second nucleotide of rpl2 CDS due to RNA editing. [file Image_4.jpeg]

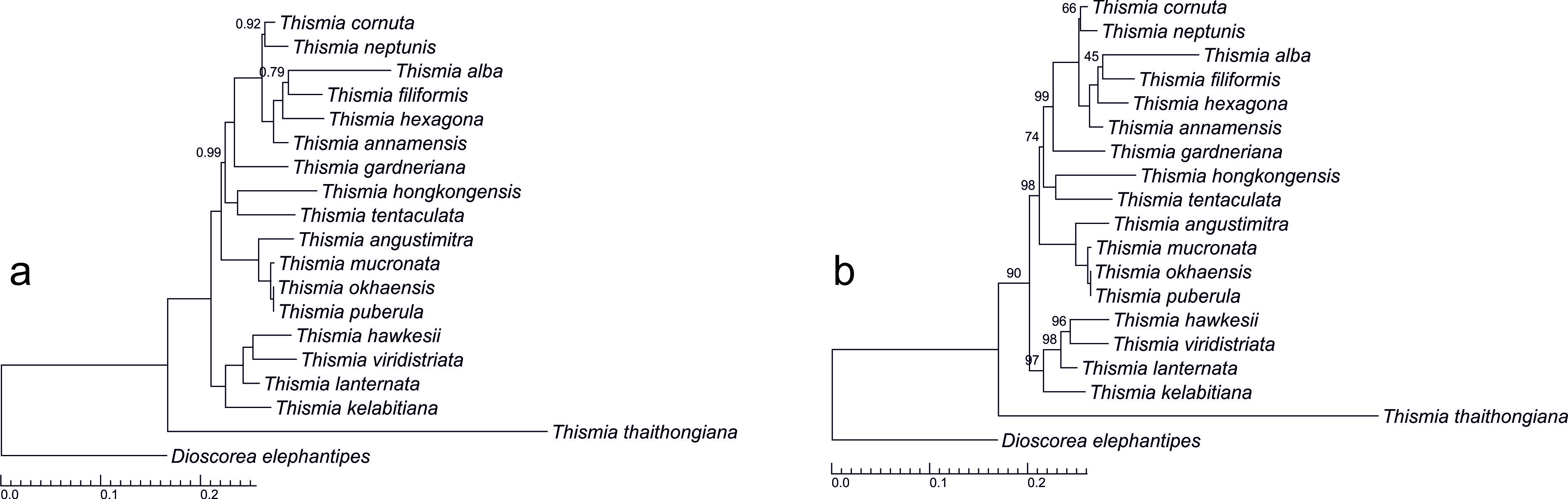

Supplement: Supplementary Figure 5 — Phylogenetic trees based on plastid genes of Thismia, (A) Bayesian (MrBayes) and (B) maximum likelihood (RAxML) analyses. Only posterior probabilities less than 1 and bootstrap support values less than 100 are shown. [file Image_5.jpeg]
